# Characterizing racial disparities in follow-up care after open-access colonoscopy

**DOI:** 10.1016/j.igie.2025.08.001

**Published:** 2025-08-11

**Authors:** Alexandria Lenyo, Kyle S. Liu, Kasey Hornbuckle, Dayna S. Early, Jean Wang, Cassandra D.L. Fritz

**Affiliations:** 1 Department of Medicine, Vanderbilt University, Nashville, Tennessee, USA; 2 Department of Medicine, Washington University School of Medicine, St. Louis, Missouri, USA; 3 Division of Gastroenterology, Department of Medicine, Washington University School of Medicine, St. Louis, Missouri, USA; 4 Alvin J. Siteman Cancer Center, Washington University School of Medicine, St. Louis, Missouri, USA

## Abstract

**Background and Aims:**

Open-access (OA) colonoscopy expands colorectal cancer (CRC) screening services. Although CRC screening disparities have been established, disparities in the completion of follow-up care after an OA colonoscopy are unknown.

**Methods:**

A retrospective cohort study included patients who had an OA colonoscopy in 2019 at a large nonprofit academic hospital in St Louis, Missouri, United States. Sociodemographic and clinical data were collected for patients who were given a short follow-up interval (<3 years) after the initial OA colonoscopy. The primary outcome was the odds of receiving follow-up care on the basis of sociodemographic factors. Multivariable logistic regressions were used to estimate adjusted odds ratios and 95% confidence intervals (CIs).

**Results:**

Of 2627 patients, 542 (20.6%) received a short-interval follow-up recommendation (mean age 59.8 [standard deviation 8.6 years]; 46.5% female). Most (57.0%) patients identified as a racial minority, with 93.9% (290 of 309) identifying as Black. Only 45.6% (247 of 542) of patients received their recommended short-interval follow-up care. White patients were more likely to receive follow-up care than patients identifying as a racial minority (52.8% vs 40.1%; *P* = .007). After adjustment for confounders, Black patients were 39% less likely to receive the appropriate follow-up for any recommendation that was <3 years (odds ratio, 0.61; 95% CI, 0.41-0.90).

**Conclusions:**

Minority patients undergoing OA colonoscopy were less likely to receive the recommended short-interval (<3 years) follow-up. Our study suggests that implementing follow-up strategies after OA colonoscopy may be imperative to address disparities in CRC screening and surveillance.

## Introduction

Endoscopic procedures continue to be in high demand within health systems. Health systems increasingly are offering open-access (OA) colonoscopy to address the need for endoscopic services. OA endoscopy is the practice whereby primary care providers order endoscopic procedures for patients without an established relationship or previous consultation from a gastroenterologist. OA colonoscopy can provide efficient and prompt care for patients with uncomplicated gastrointestinal issues and improve access to colorectal cancer (CRC) screening.^1^^,^^2^ OA colonoscopy may reduce health care costs by decreasing gastroenterology office visits, which saves patients the cost of the visit and the lost income from missing work.^2^, ^3^, ^4^ Given these benefits, OA colonoscopy has been increasingly offered in the United States since the 1990s.^3^ A 2016 analysis of 842,849 patients who underwent screening colonoscopy found that only 30% of patients had an office visit with a gastroenterologist before the procedure, whereas 70% were open access.^5^

Guidelines for OA endoscopy highlight several pertinent issues, including appropriateness of referral, patient preparedness, and assurance of follow-up. These guidelines stress that primary care providers referring patients for an OA colonoscopy should understand indications for this procedure and counsel patients on why it is recommended. It is also recommended that gastroenterologists share the procedure results and associated management recommendations with the patient and the primary care provider to ensure appropriate follow-up care.^6^

The American Society for Gastrointestinal Endoscopy guidelines highlight the importance of follow-up after OA colonoscopy, which could include recommendations for repeat colonoscopy, diagnostic imaging or laboratory tests, or a referral to a specialist, such as a colorectal surgeon.^6^ However, only a few studies have documented compliance with follow-up, to our knowledge. A retrospective cohort study of 168 patients in Cleveland, Ohio, United States, documented high rates of follow-up after OA colonoscopy, noting 75% compliance for diagnostic recommendations and 90% for therapeutic recommendations. However, this study, conducted in 2003, included a small sample of internal referrals with minimal patient demographic data, providing a limited sociodemographic landscape of OA colonoscopy follow-up.^7^ A 2018 study surveyed primary care providers across the United States regarding responsibility for arranging follow-up care after OA colonoscopy. In this study, 54% of 210 respondents stated that primary care providers were responsible for ensuring proper follow-up care in their practice, and 34% reported that endoscopists were responsible.^8^

Currently, it is unknown whether disparities exist in follow-up care after OA colonoscopy, especially for patients with high-risk OA colonoscopy findings. To address this knowledge gap, we explored the association between sociodemographic factors and completion of follow-up care for patients who used the OA system for colonoscopy at a large nonprofit tertiary academic referral center in St Louis, Missouri, United States. St Louis city and county are racially and socioeconomically diverse metropolitan areas. The city of St Louis is 45.7% White and 43.1% Black, with a median household income of $55,279 in 2023. The county is 66.9% White and 25.3% Black, with a greater reported median income of $81,340.^9^ The diverse socioeconomic and racial characteristics of the greater St Louis metropolitan area allow for a unique environment for studying health care disparities.

## Methods

### Study design

We conducted a retrospective cohort study using all OA colonoscopies performed in 2019 at Barnes-Jewish Hospital (BJH). BJH is a large (>1200 bed) nonprofit hospital in St Louis. BJH is the largest hospital within the Barnes-Jewish Hospital Health Care System, which includes a catchment area of community hospitals and clinics in Illinois and Missouri. Chart review was completed between August 2022 and January 2024. This study was submitted to the Washington University School of Medicine Institutional Review Board and found exempt. We followed the Strengthening the Reporting of Observational Studies in Epidemiology reporting guidelines.

OA colonoscopy at BJH is an established system that annually includes more than 2000 OA colonoscopies. A primary care provider places the order for an OA colonoscopy. An attending gastroenterologist provides OA colonoscopy services with or without a gastroenterology fellow. Procedural documentation and postprocedure recommendations are provided to the patient and their primary care provider after the procedure. For any necessary follow-up after endoscopy, nurses set an electronic health record reminder 3 months before the date of required follow-up. At this time, the patient and the primary care provider receive a message or letter to remind them to schedule the necessary follow-up. Unless patients establish care with a gastroenterologist during the follow-up interval, orders for follow-up colonoscopies are placed by the patient's primary care provider.

### Study population

The electronic medical record was queried to provide patients who underwent endoscopy at BJH in 2019. Patients were included if they used the OA system, underwent colonoscopy, and were 18 years or older at the time of endoscopy. We excluded patients who died before the 3-year follow-up interval. If a patient had more than 1 OA colonoscopy during the study period (3 years after endoscopy in 2019), only the initial colonoscopy was included.

Demographic information was collected from the electronic medical record, including age at endoscopy, sex, self-identified race, self-identified ethnicity, insurance status, and type. Clinical characteristics, including personal history of CRC, first-degree family history of CRC, previous polyp history, smoking status, and Charlson Comorbidity Index score, also were collected. All procedures were performed with the patients under propofol-based monitored anesthesia care. Procedural data included endoscopy indication, endoscopic findings, pathology, and Boston Bowel Preparation Scale (BBPS) score. A BBPS score of 6 or greater was considered adequate bowel preparation for the purposes of this study. Follow-up recommendations and time to recommended follow-up, as determined by the endoscopist, were also collected. All forms of recommended follow-up were grouped together for the primary outcome analysis.

### Statistical analysis

We evaluated the association between each sociodemographic or clinical factor and completion of follow-up care for patients who received a short follow-up interval, defined as needing follow-up 3 years or less after initial OA colonoscopy. Means (standard deviation) were used to express continuous variables. Means were compared using *t* tests. Categorical variables were compared using χ^2^. Multivariable logistic regression models were used to estimate odds ratios (ORs) and 95% confidence intervals (CIs). We adjusted for age (continuous), sex (male/female), smoking status (current/prior/never), personal history of CRC (yes/no), family history of CRC (yes/no), higher CRC risk (yes/no), insurance type (private/nonprivate [Medicare, Medicaid], none), and recommended follow-up interval (<12 months/≥12-36 months). All statistical analyses were completed using SAS 9.4 (SAS Institute, Cary, NC, USA). A 2-sided *P*-value of <.05 was deemed statistically significant.

## Results

**Figure 1:**
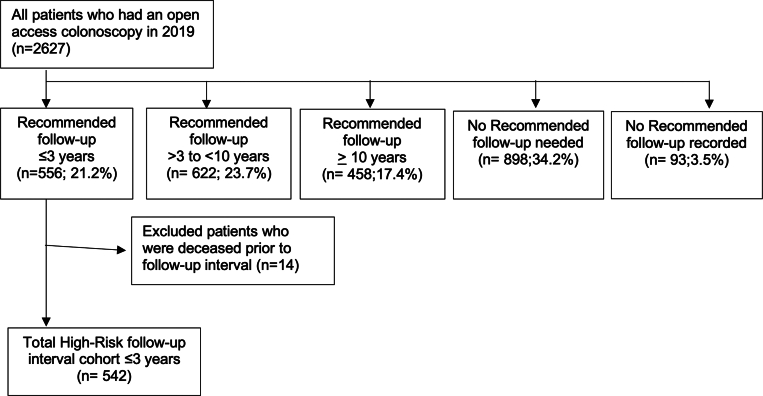
Flow diagram for the study cohort.

A total of 2627 patients underwent OA colonoscopy. The mean age was 58.7 (9.2) years, and 55.2% (n = 1450) were female. Of the total OA colonoscopy cohort, 1606 (61.1%) patients self-identified as minority, and 93.6% (1503/1606) of these patients identified as Black. The recommended follow-up time after OA colonoscopy was 10 years for 458 patients (17.4%), more than 3 years but less than 10 years for 622 patients (23.7%), and 3 years or less for 556 patients (21.2%). No follow-up was needed for 898 (34.2%) or recorded for 93 (3.5%) patients. Among the 556 patients who received a short follow-up interval (≤3 years), 14 patients died before their recommended follow-up (Fig. 1).

A total of 542 patients who underwent an OA colonoscopy were included in the short follow-up interval cohort, with an average age of 59.8 (8.6) years. This cohort was 46.5% female, and 43% of patients identified as White (Table 1). Most patients underwent OA colonoscopy for a screening/surveillance indication (83.8%), with 31.2% of patients having a previous adenoma. Adequate bowel preparation was documented in 78.8% of patients. The most common pathology was tubular adenoma (56.3%), followed by serrated polyps (12.2%) and tubulovillous adenoma (4.8%). Seven adenocarcinomas were documented through OA colonoscopy. The main follow-up care recommendation was future colonoscopy (91.5%), whereas a small portion of patients were recommended to undergo imaging (2.8%) or were referred to oncology or colorectal surgery (2.4%) as a result of colonoscopy findings (Table 2). Less than one-half (45.6%; 247 of 542) of patients who were given a short follow-up interval were able to complete the recommended follow-up. Of patients recommended to complete a colonoscopy, completion was only 41.7%.

**Table 1:** Demographic characteristics of patients undergoing open-access colonoscopy who required follow-up in 3 years or less, Barnes-Jewish Hospital, 2019

Characteristics	All patients n = 542 (%)	Follow-up not completed n = 295 (%)	Follow-up completed n = 247 (%)
Age at endoscopy, years, mean (SD)	59.8 (8.6)	59.7 (8.2)	59.9 (9.1)
Sex			
Male	290 (53.5)	154 (52.2)	136 (55.1)
Female	252 (46.5)	141 (47.8)	111 (44.9)
Race∗			
White	233 (43.0)	110 (37.3)	123 (49.8)
Black or African American	290 (53.5)	172 (58.3)	118 (47.8)
Asian	12 (2.2)	8 (2.7)	4 (1.6)
Other†	7 (1.3)	5 (1.7)	2 (0.8)
Hispanic ethnicity∗	7 (1.3)	1 (0.3)	6 (2.4)
Smoking status			
Never	223 (41.1)	111 (37.6)	112 (45.3)
Former smoker	143 (26.4)	76 (25.8)	67 (27.1)
Current smoker	176 (32.5)	108 (36.6)	68 (27.5)
Private insurance	233 (43.0)	123 (41.7)	110 (44.5)
Nonprivate insurance	309 (57.0)	172 (58.3)	137 (55.5)
Medicare	153 (28.2)	77 (26.1)	76 (30.8)
Medicaid	91 (16.8)	57 (19.3)	34 (13.7)
Gateway to Better Health‡	46 (8.5)	30 (10.2)	16 (6.5)
None	19 (3.5)	8 (2.7)	11 (4.5)
Charlson Comorbidity Score, mean (SD)	1.8 (2.4)	1.7 (2.4)	1.9 (2.4)
Previous adenomas	169 (31.2)	87 (29.5)	82 (33.2)
Personal history of CRC	22 (4.1)	9 (3.1)	13 (5.3)
Family history of CRC	57 (10.5)	32 (10.8)	25 (10.1)

*CRC*, Colorectal cancer; *SD*, standard deviation.

∗ Denotes statistical significance between groups (follow-up not completed vs completed).

† Other: individuals who identify as multiracial, Pacific Islander, or unknown.

‡ Gateway to Better Health: Health program for uninsured individuals in St Louis city or St Louis County, Missouri, United States, with income at or below 100% of the federal poverty level, but who do not qualify for Medicaid or Medicare. This program enables participants to receive care at designated health care centers.

**Table 2:** Procedural characteristics of patients undergoing open-access colonoscopy who required follow-up in 3 years or less, Barnes-Jewish Hospital, 2019

Characteristics	All patients n = 542 (%)	Follow-up not completed n = 295 (%)	Follow-up completed n = 247 (%)
Indication			
Screening/surveillance	454 (83.8)	254 (86.1)	200 (81.0)
Diagnostic	88 (16.2)	41 (13.9)	47 (19.0)
Boston Bowel Prep Score ≥6∗	424 (78.2)	244 (82.7)	180 (72.9)
Pathology			
Tubular adenoma	305 (56.3)	177 (60.0)	128 (51.8)
Tubulovillous	26 (4.8)	12 (4.1)	14 (5.7)
Serrated	66 (12.2)	39 (13.2)	27 (10.9)
Adenocarcinoma	7 (1.3)	1 (0.3)	6 (2.4)
Other cancer	3 (0.6)	0	3 (1.2)
Recommended follow-up interval∗			
<12 months	235 (43.4)	87 (29.5)	148 (59.9)
12-36 months	307 (56.6)	208 (70.5)	99 (40.1)
Follow-up care recommended			
Colonoscopy	496 (91.5)	289 (98.0)	207 (83.8)
Colorectal surgery or oncology	13 (2.4)	2 (0.7)	11 (4.5)
CT or MRE	15 (2.8)	1 (0.3)	14 (5.7)
Other endoscopy	2 (0.4)	0	2 (0.8)
Laboratory tests	2 (0.4)	0	2 (0.8)

*CT*, Computed tomography; *MRE*, magnetic resonance enterography.

∗ Statistical significance between groups (follow-up not completed vs completed).

**Supplementary Figure 1:**
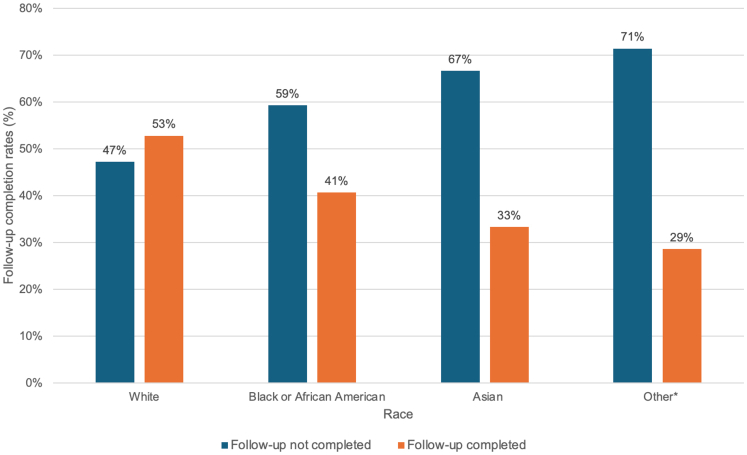
Open access colonoscopy follow-up completion by race. Percentages represent the proportion of individuals within each racial group who completed or did not complete follow-up. ∗Other: individuals who identify as multiracial, Pacific Islander, or unknown.

**Supplementary Figure 2:**
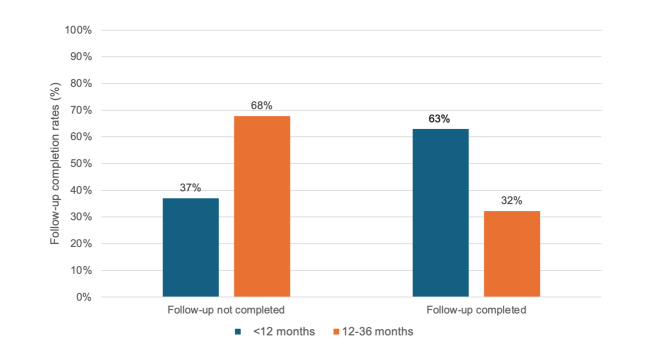
Open access colonoscopy follow-up completion by recommended follow-up interval. Percentages represent the proportion of individuals within each recommended follow-up interval (<12 months vs 12 and <36 months) who completed or did not complete follow-up.

Among the patients who were given a follow-up interval of less than 12 months, 39% (92 of 235) were less than 1 month. Of these 92 patients, 65 were given this follow-up interval as the result of poor bowel preparation (BBPS score of less than <6). Patients who were unable to complete the recommended follow-up care were more likely to be a racial minority, current smoker, have nonprivate insurance, and have a follow-up recommendation between 12 and 36 months (Supplementary Figs. 1 and 2, available online at www.igiejournal.org).

Multivariate analysis was conducted to investigate factors associated with the completion of follow-up care after OA colonoscopy (Table 3). When compared with White patients, Black race was found to be negatively associated with completion of timely follow-up (OR, 0.61; 95% CI, 0.41-0.91). Similarly, when compared with an interval of <12 months, a follow-up recommendation of 12 months to 36 months was negatively associated with the completion of follow-up care (OR, 0.25; 95% CI, 0.16-0.39). No statistically significant association was found for completion of follow-up care with sex, family history of CRC, or type of insurance.

**Table 3:** Factors associated with completion of follow-up care after open-access colonoscopy

	Follow-up not completed n (%)	Follow-up completed n (%)	Univariate OR (95% CI)∗	Multivariate OR (95% CI)†
Sex				
Male	154 (28.4)	136 (25.1)	Reference	Reference
Female	141 (26.0)	111 (20.5)	0.97 (0.67-1.39)	0.97 (0.65-1.44)
Race				
White	110 (20.3)	123 (22.7)	Reference	Reference
Black	172 (31.7)	118 (21.8)	**0.61 (0.43-0.87)**	**0.61 (0.41-0.90)**
Family history				
No	263 (48.5)	222 (41.0)	Reference	Reference
Yes	32 (5.9)	25 (4.6)	0.91 (0.52-1.61)	0.90 (0.49-1.64)
Insurance type				
Private	123 (22.7)	110 (20.3)	Reference	Reference
Medicare/Medicaid	172 (31.7)	137 (25.3)	0.93 (0.64-1.35)	0.92 (0.61-1.38)
Interval				
<12 months	87 (16.1)	148 (27.3)	Reference	Reference
12-36 months	208 (38.4)	99 (18.3)	**0.27 (0.19-0.39)**	**0.25 (0.16-0.39)**

Bold text highlights statistically significant values.

*CI*, Confidence interval; *OR*, odds ratio.

∗ Adjusted for age and sex.

† Adjusted for age (continuous), sex (male/female), race (White/Black), smoking status (never, former, current), personal history of colorectal cancer (yes/no), family history of colorectal cancer (yes/no), pathology (tubular adenoma, tubulovillous, serrated lesion, adenocarcinoma, other cancer), interval (<12 months/12-36 months), adequate prep (Boston Bowel Preparation Scale ≥ 6; yes/no), and insurance type (private, Medicare/Medicaid).

## Discussion

Our findings reveal significant gaps in adherence to follow-up care after OA colonoscopy, particularly among patients recommended to return within 12 to 36 months. More than half (54.4%) of patients failed to complete follow-up. Notably, most (73%) had adequate bowel preparation, suggesting that barriers to adherence extend beyond procedural issues. Follow-up completion after OA colonoscopy varied by sociodemographic factors. Black patients, current smokers, patients lacking private insurance, and patients with longer follow-up intervals were less likely to return. After adjusting for confounders, Black patients were 39% less likely than White patients to receive timely follow-up, highlighting persistent inequities in postcolonoscopy care. Furthermore, longer surveillance intervals (12-36 months) were strongly associated with noncompletion, suggesting that patient engagement diminishes as the interval to follow-up lengthens. The low follow-up completion rate among the entire cohort (45.5%) is also a key result. Our findings underscore the need for targeted interventions to improve follow-up adherence, particularly among vulnerable populations and for longer surveillance intervals.

A recent study found that Black patients and patients with Medicare were associated with a 3-fold increased odds of endoscopy appointment cancellation.^10^ Similarly, greater neighborhood poverty rates have been associated with nonadherence to scheduled colonoscopy.^11^ Taken together in the context of our study, these findings highlight the need for greater intentionality in offering OA colonoscopy to appropriate patients and identifying patients who require additional support to prepare for their colonoscopy and complete the necessary follow-up. Ideally, all patients should complete follow-up after OA colonoscopy. Still, a follow-up goal of 80% is a pragmatic benchmark, similar to the American Cancer Society National Colorectal Cancer Roundtable goals.^12^ Follow-up after OA colonoscopy is vital, because being up to date with CRC screening is associated with a 62% reduction in CRC-related mortality.^13^

Regarding patient communication, a study by Staff et al^14^ found that patients undergoing OA colonoscopy are less adequately informed of the process for colonoscopy compared to patients referred from gastroenterology clinics. An additional study found that despite verbal and written communication, 28% of patients could not recall their postendoscopy recommendations,^15^ highlighting the need for improved documentation and communication of endoscopy results.^7^ Our study contributes to the literature by highlighting the disparities that can arise when health care processes are developed without considering sociodemographic factors and other social determinants of health. Interventions, such as automated messages and patient reminders, could be integrated to decrease the burden on patients and providers to remember OA follow-up. Atlas et al^16^ examined interventions to improve timely follow-up of overdue abnormal cancer screening results, including CRC. This study found that electronic health record reminders and patient outreach, with or without patient navigation, improved timely follow-up for overdue abnormal cancer screenings.

A strength of our study is the novel investigation of factors associated with poor follow-up after OA colonoscopy. This study used a diverse patient cohort referred from multiple clinics, thereby enhancing the generalizability of our findings. In addition, our study is one of the largest single-centered studies assessing disparities in follow-up care after OA colonoscopy, to our knowledge.

We acknowledge limitations to our study. The follow-up period after OA colonoscopy overlapped with the coronavirus disease 2019 (COVID-19) global pandemic, potentially affecting rates of follow-up. Racial disparities in health care use were well-documented during the pandemic.^17^^,^^18^ A recent study using a large national database examined the impact of the COVID-19 pandemic on CRC screenings. During the first wave (March and April 2020) of the COVID-19 pandemic, CRC screening decreased; however, screenings recovered to prepandemic levels by September 2020.^19^ In our study, significant racial disparities were observed with longer follow-up periods when pandemic effects on endoscopy were significantly less pronounced. Furthermore, our endoscopy center made concerted efforts to recapture patients who may have been lost to follow-up due to scheduling challenges.

Second, as the result of modified protocols during the study period, additional sociodemographic variables such as educational status, employment status, and economic status were not reliably collected. Third, with this retrospective observational study relying on medical record review, residual confounding and inaccuracies or omissions in initial chart documentation cannot be excluded. Lastly, although we cannot confirm we captured every follow-up endoscopy that occurred outside our health system, most patients continue to obtain care within our large integrated health care system. We investigated all records from other health care entities available to us for additional follow-up results.

Within our cohort of all patients who received OA colonoscopy in 2019 (n = 2627), only 3.5% of patients had missing follow-up data, and 34.2% of patients were informed that follow-up was not required. Most patients who did not require follow-up were elderly individuals whose age placed them outside the U.S. Preventive Services Task Force's recommended screening age.^20^ Most patients without a recorded follow-up plan underwent a diagnostic procedure, and high-risk findings were not identified.

Our study further highlights the ongoing need for intervention strategies that can target highly vulnerable patient populations. A recent study found that, in an African American study population, community-based, in-person educational sessions significantly improved rates of CRC screening.^21^ A systematic review of interventions to increase follow-up of abnormal CRC screening results in safety net settings revealed patient navigation interventions that provide patient education, reminders, and assistance with scheduling colonoscopies showed promising improvements in follow-up.^22^ A 2021 study found that clinics with higher rates of successful follow-up after an abnormal CRC screening used registries to track patients needing follow-up until their colonoscopies were performed.^23^ The electronic health record could be better leveraged to create these workflows and registries. Further research on interventions to improve patient preparedness and follow-up rates for all demographics after OA colonoscopy is greatly needed, with additional focus on creating intentional strategies for the most vulnerable patient populations.

## Conclusion

This study identified low rates of appropriate and timely follow-up care after an initial OA colonoscopy. Black race and a recommended follow-up interval between 12 and 36 months were associated with lower rates of adequate follow-up. Interventions to build operational systems that are better able to identify and target patients who require additional support are needed to ensure that all patients are able to receive follow-up care after OA colonoscopy.

## Data availability

Data are available from the corresponding author upon reasonable request.

## Patient Consent

This article does not discuss individual patients, so no consent was needed.

## Disclosure

The following authors disclosed financial relationships: D. Early: Scientific Advisory Board for Guardant Health. A. Lenyo: Stock in 10.13039/100006483AbbVie and 10.13039/100001316Abbott Laboratories. All other authors disclosed no financial relationships. This work was supported by P30 DK052574 and the Faculty Diversity Scholars Award from Washington University in St Louis. This content is solely the authors' responsibility and does not necessarily represent the official views of the 10.13039/100000002National Institutes of Health.
